# Targeting Cancer Through Thymoquinone: From Molecular Mechanisms to Clinical Prospects

**DOI:** 10.3390/ijms262211029

**Published:** 2025-11-14

**Authors:** Nosayba Al-Damook, Molham Sakkal, Mostafa Khair, Walaa K. Mousa, Ghalia Khoder, Rose Ghemrawi

**Affiliations:** 1 College of Pharmacy, Al Ain University, Abu Dhabi P.O. Box 112612, United Arab Emirates; nosayba.aldamook@aau.ac.ae (N.A.-D.); molham.sakkal@gmail.com (M.S.); walaa.mousa@aau.ac.ae (W.K.M.); 2 AAU Health and Biomedical Research Center, Al Ain University, Abu Dhabi P.O. Box 112612, United Arab Emirates; 3 Core Technology Platforms, New York University Abu Dhabi, Abu Dhabi P.O. Box 129188, United Arab Emirates; mrk6@nyu.edu; 4 Department of Pharmaceutics and Pharmaceuticals Technology, College of Pharmacy, University of Sharjah, Sharjah P.O. Box 27272, United Arab Emirates; gkhoder@sharjah.ac.ae; 5 Sharjah Institute for Medical Research, University of Sharjah, Sharjah P.O. Box 27272, United Arab Emirates

**Keywords:** Thymoquinone, cancer therapy, chemotherapy resistance, combination therapy, anticancer agents

## Abstract

Thymoquinone (TQ), the active compound in Nigella sativa (black seed), has shown promising effects against cancer in many laboratory studies. In this review, we explore how TQ works on different aspects of cancer, from stopping cancer cell growth and spread, to triggering cancer cell death, reducing inflammation, and helping the immune system fight back. We also highlight how TQ may overcome one of the biggest problems in cancer treatment—chemoresistance. When used together with common treatments like chemotherapy, radiation, or immunotherapy, TQ has been shown to improve their effects and reduce harmful side effects in preclinical models. Our review further discusses how TQ affects cancer stem cells, the tumor environment, and gene regulation through epigenetics. While these findings are encouraging, the lack of human studies remains a major gap. We also address TQ’s limited absorption and suggest ways to improve its delivery in the body, such as using nanoparticles or other carriers. Through this review, we aim to show the wide-ranging potential of TQ as a natural compound that may help make cancer treatments more effective and better tolerated. We call for clinical studies to take this research further and bring TQ closer to use in real-world cancer care.

## 1. Introduction

Despite significant advancements in oncology, the global burden of cancer continues to escalate, underscoring the critical need for innovative and effective treatment approaches [1]. According to the American Cancer Society’s 2025 statistics, over two million new cancer cases are anticipated in the United States alone, with more than 600,000 related deaths expected this year [2].

The development of new cancer therapies faces numerous challenges, primarily stemming from the aggressive and complex nature of cancer cells, characterized by rapid proliferation, local tissue invasion, and high metastatic potential. These traits demand early detection and targeted therapeutic strategies [3,4]. Chemotherapy remains a cornerstone in cancer management due to its efficacy in eliminating malignant cells [5]. Nevertheless, chemotherapy’s widespread use is significantly hampered by its non-specific cytotoxicity, which adversely affects both cancerous and healthy cells. This lack of selectivity results in severe side effects, including hair loss, immunosuppression, gastrointestinal distress, and organ toxicity, ultimately diminishing patients’ quality of life.

Given these limitations, alternative and complementary therapeutic strategies, especially those derived from natural sources, have garnered increased attention. Phytochemicals and bioactive compounds extracted from medicinal plants have demonstrated promising anticancer potential, often exhibiting selective cytotoxicity and fewer side effects compared to conventional chemotherapy [6]. Among these natural compounds, Thymoquinone (TQ), the primary bioactive component of Nigella sativa (black seed), has emerged as particularly noteworthy due to its multi-targeted mechanisms of action against cancer [7].

**Figure 1:**
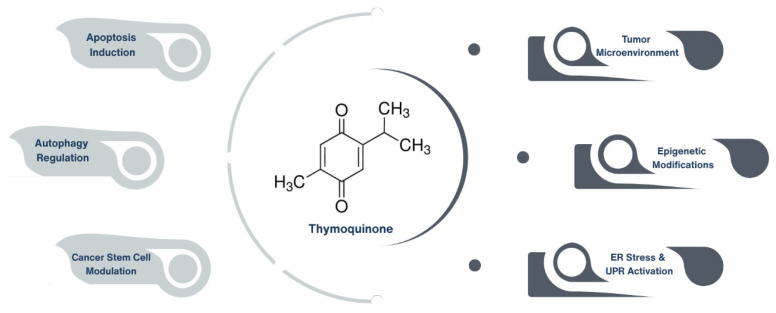
Emerging anticancer mechanisms of thymoquinone in preclinical studies. Thymoquinone, the primary bioactive constituent of Nigella sativa, exerts anticancer effects through multiple mechanisms, including apoptosis induction, autophagy regulation, and modulation of cancer stem cells. Additionally, it impacts the tumor microenvironment, influences epigenetic modifications, and activates endoplasmic reticulum (ER) stress and unfolded protein response (UPR) pathways.

Emerging preclinical research highlights TQ’s potent anticancer properties, including apoptosis induction [8], autophagy regulation [9], endoplasmic reticulum (ER) stress [10,11], unfolded protein response (UPR) modulation [12], modulation of cancer stem cells (CSCs) [13], interactions within the tumor microenvironment (TME) [14], and epigenetic modifications [15]. Furthermore, TQ has shown considerable promise in combination therapy approaches, where it enhances the efficacy of conventional chemotherapeutic agents while mitigating their associated toxicities [13] (Figure 1).

While previous reviews have summarized TQ’s anticancer potential, comprehensive coverage integrating detailed molecular mechanisms, effects on CSCs, TME modulation, epigenetic impact, and strategies for overcoming drug resistance through combination therapy remains lacking. Moreover, significant gaps persist regarding how TQ’s complex molecular actions translate to clinical outcomes and how advanced drug-delivery systems might optimize its therapeutic efficacy.

This review aims to comprehensively analyze TQ’s therapeutic potential in cancer therapy, focusing on its molecular mechanisms of action, role in overcoming drug resistance, and potential clinical applications. Additionally, we critically examine recent advances (2023–2025) in TQ-related cancer research, particularly its emerging roles in epigenetic regulation, immune modulation, and combination therapies. Furthermore, the review identifies existing challenges, including bioavailability limitations and the need for clinical validation, and proposes clear recommendations for future translational studies. Ultimately, this detailed evaluation aims to inform future research directions and support TQ’s clinical development as a viable, personalized cancer therapeutic.

## 2. Molecular Mechanisms of Thymoquinone in Cancer

**Figure 2:**
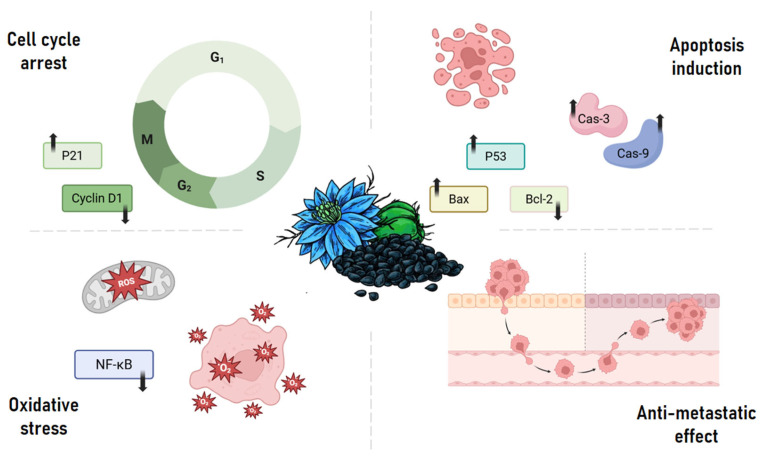
Key molecular mechanisms underlying the anticancer activity of thymoquinone. Thymoquinone exerts its anticancer effects by targeting multiple pathways: it induces cell cycle arrest through modulation of cyclin D1 and upregulation of p21; promotes apoptosis via p53 activation, Bax upregulation, Bcl-2 suppression, and caspase-3/9 activation; inhibits metastasis by interfering with cancer cell invasion and migration; and triggers oxidative stress by downregulating NF-κB signaling, leading to reactive oxygen species (ROS) accumulation.

The anticancer properties of Thymoquinone (TQ) arise from its complex interactions with multiple oncogenic pathways. TQ exhibits diverse biological activities including anti-inflammatory, antioxidative, anti-angiogenic, and anti-metastatic effects, as well as the induction of apoptosis and regulation of multiple tumor suppressor genes. The compound primarily inhibits tumor cell proliferation through mechanisms such as cell cycle arrest, disruption of microtubule organization, and downregulation of cell survival proteins [16]. Furthermore, TQ’s proapoptotic activity involves mitochondrial dysfunction and caspase activation. It also modulates epithelial–mesenchymal transition (EMT) and targets cancer stem cells (CSCs), reducing metastatic potential and hindering cancer progression through its antioxidative properties [17] (Figure 2).

Specifically, TQ induces cell cycle arrest by blocking the activation of cyclin E and cyclin D and up-regulating cell cycle inhibitors p27 and p21, causing G1 phase arrest in breast, colon, and osteosarcoma cancer cells. Interestingly, the concentration of TQ determines the cell cycle phase affected; high concentrations induce G2 phase arrest, while low concentrations lead to S phase arrest in breast cancer cells (MCF-7) [16]. These pleiotropic actions make TQ a promising multi-targeted therapeutic agent, particularly in the era of personalized medicine and systems oncology. Moreover, its selective toxicity toward malignant cells positions it as a safer alternative or adjuvant to conventional chemotherapeutics.

### 2.1. Apoptosis Induction

TQ can induce apoptosis by several mechanisms. In breast cancer cell line MCF-7, it was found that TQ upregulates the expression of p53 in a time-dependent manner, thus promoting apoptosis and inhibiting the proliferation of these cells [18]. TQ was also reported to induce apoptosis in HCT116 human colon cancer cells by enhancing the expression of the pro-apoptotic Bax (BCL-2 associated X) protein while downregulating the anti-apoptotic Bcl-2 (B-cell lymphoma-2) and Bcl-xL proteins [19]. Similarly, TQ treatment caused a significantly higher expression of p53 and p21 and downregulated that of Bcl-2 in human pancreatic ductal adenocarcinoma (PDAC) AsPC-1 and MiaPaCa-2 cell lines. This was further confirmed in vivo, where TQ was able to significantly reduce tumor size in human PDAC xenograft models [20]. In contrast, a study investigating TQ’s activity in models of aggressive prostate cancers (androgen receptor (AR)-independent (C4-2B) and AR naïve (PC-3) prostate cancer cells) showed that in this cellular context, TQ did not increase the activity of caspases, but it significantly upregulated the expression of apoptosis-inducing factor-1, and downregulated the expressions of several Bcl2-related proteins, such as BAG-1, Bcl2, Bcl2A1, Bcl2L1, and BID [21]. It was also shown that TQ can induce p53-dependent apoptosis in osteosarcoma. In a study comparing normal human osteoblasts, p53-null MG63 osteosarcoma cells, and mutant-p53 MNNG/HOS osteosarcoma cells, TQ reduced cell viability more significantly in MG63 cells. Flow cytometric analysis revealed a time-dependent increase in the PreG1 cell population, indicating enhanced apoptosis, particularly in MG63 cells. DNA fragmentation assays (ELISA and DASH) further confirmed the apoptotic effects of TQ, with a marked increase in cytoplasmic histone-associated DNA fragments in MG63 cells but minimal changes in MNNG/HOS cells. Mechanistically, TQ-induced apoptosis in MG63 cells was associated with the activation (cleavage) of caspase-3 and caspase-9, suggesting the involvement of the intrinsic (mitochondrial) apoptotic pathway. Interestingly, although both MG63 and MNNG/HOS cells showed modulation of Bax/Bcl-2 protein ratios following TQ treatment, apoptosis was predominantly observed in MG63 cells, indicating that caspase activation, rather than Bax/Bcl-2 regulation, was the primary driver of TQ-induced cell death. These findings suggest that TQ more effectively induces apoptosis through the mitochondrial caspase-dependent pathway in p53-null cancer cells compared to cells harboring mutant p53 [22]. Importantly, these findings indicate that TQ’s pro-apoptotic actions are not limited to a single mechanism but involve multiple parallel and redundant death pathways, including mitochondrial, p53-dependent, and caspase-independent routes. Another study examining the effect of TQ on SaOS-2 osteosarcoma cells using FITC-conjugated Annexin V and PI staining showed that TQ treatment increased the percentage of early apoptotic cells in a dose-dependent manner. This indicates that TQ’s cytotoxic effect on these cells may be linked to inducing apoptosis [23]. Very interestingly, this natural compound is able to induce these apoptotic effects in cancer cells alone without having a significant effect on normal cells. A study examining the effects of TQ on glioblastoma vs. hTERT immortalized human foreskin fibroblasts revealed that the expression of pro-apoptotic Bax protein level increased only in glioblastoma cells (M059K and M059J) following TQ treatment but not in the fibroblasts. Moreover, TQ increased the level of cytosolic cytochrome c, which is critical for the initiation of apoptosis, and induced cell death through apoptosis as indicated by the increase in cell population showing positive staining for Annexin V [24]. Investigations on HeLa cervical cancer cells demonstrated that TQ promoted apoptosis by modulating the expression of apoptosis-related genes. Specifically, TQ upregulated the expression of pro-apoptotic genes such as BCL2L10, BIK (BCL-2 interacting killer), caspase-1, and FASL. On the other hand, it downregulated genes associated with anti-apoptotic functions mediated by NF-κB signaling, including BID, BIK, RELA, RELB, TNF, TNFRSF10A, TNFRSF10B, and TRAF3 [25].

### 2.2. Cell Cycle Arrest

Other than inducing apoptosis, TQ is able to downregulate cyclin D1/CDK4, halting cell division. In the A549 lung cancer cell line, TQ treatment was shown to downregulate cyclin D1, MMP2, MMP9, and PCNA (proliferating cell nuclear antigen). Moreover, it was shown to block the phosphorylation of ERK1/2, leading to an overall inhibitory effect on the proliferation, migration, and invasion of A549 cancer cells [26]. In the context of human hepatocellular carcinoma, HepG2 cells treated with TQ for 12 or 18 h showed that TQ caused a significant accumulation of cells in the G2/M-phase. It was proposed that the observed G2 arrest is due to the induction of G2 checkpoint machinery that allows damaged DNA to be repaired before cells move to the next cell cycle stage. However, if DNA damage is extensive, cell death by apoptosis will commence [27]. Similar effects were shown in hepatocellular carcinoma where TQ suppresses G2/M cell cycle [27]. Furthermore, TQ treatment has been shown to downregulate proteins modulated by E2F-1 which are essential for cell cycle progression [28]. This effect was observed in LNCaP prostate cancer cells, in which TQ treatment was found to reduce androgen receptor (AR) and E2F-1-associated proteins, arrest the G1 to S phase transition of cancer cell cycles, and substantially increase the level of p21Cip1 (cyclin-dependent kinase inhibitor 1), p27Kip1 (cyclin-dependent kinase inhibitor 1B), and Bax [29]. With regard to consistency, TQ was found to induce similar effects in HL-60 leukemia cells, where it induced cell cycle arrest at G1 and S phases [30]. In canine osteosarcoma COS31 cells, flow cytometry showed that TQ caused a concentration-dependent decrease in the number of COS31 cells in the S-phase and an increase in the number of cells in the G1-phase. This effect was observed after 24 h or 48 h treatment but not with shorter incubation times. This observed cell cycle arrest at G1 correlated well with the induction of apoptosis, which was also reported in this study to be time- and concentration-dependent. This study concluded that TQ activates Gl-phase checkpoint shortly after treatment, and the cells either progress through the cell cycle or undergo apoptosis [31]. Another study on osteosarcoma showed that TQ caused a more significant increase in PreG1 cell population in p53-null MG63 osteosarcoma cells compared to mutant-p53 MNNG/HOS cells. This increase in PreG1 cell population in MG63 was accompanied by a decrease in the percentage of cycling cells in S and G2/M phases. On the other hand, TQ induced G2/M arrest in MNNG/HOS cells which was associated with upregulation of p21WAF1 protein expression [22]. Similarly, TQ induced G1 phase cell cycle arrest and apoptosis in MDA-MB-231 and MDA-MB-468 triple negative breast cancer cells with mutant p53. This study also showed that TQ inhibited TNBC cell growth without affecting that of normal human mammary epithelial cells [32].

Investigations on human multiple myeloma cell lines U266 and RPMI 8226 revealed that TQ downregulates the expression of the cell cycle regulator cyclin D1, as well as the anti-apoptotic proteins Bcl-2, Bcl-xL, survivin, and Mcl-1, and the angiogenic gene product VEGF. These effects were time-dependent and more prominent with longer incubation duration. Since D-type cyclins are essential for the progression of cells from the G1 phase of the cell cycle to the S phase, the rapid decline in cyclin D1 levels that was noticed in TQ-treated cells was associated with an increased accumulation of the cell population in the sub-G1 phase, which is indicative of apoptotic cell death [33]. A similar result was found in glioblastoma cells, where more cells were entrapped in the sub-G1 phase of the cell cycle following treatment. Moreover, reduction in cell viability was attributed to the induction of apoptosis, as evidenced by a higher percentage of cells with Annexin V staining [24].

Collectively, TQ exerts a phase-specific and context-dependent arrest of the cancer cell cycle, often in conjunction with apoptotic priming. This dual action enhances its therapeutic appeal, especially in tumors with deregulated cyclin-dependent kinases or disrupted checkpoint fidelity.

### 2.3. Anti-Metastatic and Anti-Angiogenic Effects

Several studies have confirmed the anti-migratory effects of TQ in different types of cancer. For instance, Khan et al. found that TQ treatment resulted in a dose-dependent reduction in the transcription activity of TWIST1, a promoter of endothelial-to-mesenchymal transition (EMT), in BT 549 breast cancer cell line. Furthermore, TQ upregulated the expression of E-cadherin and downregulated the expression of N-cadherin genes associated with TWIST1. These results indicate the ability of TQ to inhibit cancer cell migration and invasion [34]. In another study, TQ was also found to inhibit the expression of chemokine receptor type 4 (CXCR4)—which is associated with increased cell proliferation and metastasis and is an indicator of poor prognosis in patients with breast cancer—in an MDA-MB-231 triple negative breast cancer cell line in a dose- and time-dependent manner. These anti-migratory and anti-invasiveness effects were p65-dependent, where p65 deletion reversed the observed effects. TQ was also able to inhibit tumor growth and tumor vascularity in a chick chorioallantoic membrane assay model of this cell line. Moreover, TQ treatment significantly suppressed multiple lung, brain, and bone metastases in a dose-dependent manner in a metastatic breast cancer mouse model where the H&E and immunohistochemical analysis of bone isolated from TQ-treated mice indicated a reduction in the number of osteolytic lesions and the expression of metastatic biomarkers [35]. Similar anti-metastatic properties were found in renal cell carcinoma (RCC) 786-O-SI3 cells. Although this study concluded that TQ had no significant cytotoxicity against these highly aggressive cells in contrast to findings in other types of cancers, the study highlighted a significant inhibitory effect of TQ on invasion and metastasis in the context of RCC. By performing the transwell invasion assay, the study showed that TQ significantly reduced the invasiveness of 786-O-SI3 cells. The transmigration analysis revealed that TQ significantly decreased the number of transmigrated cells in a dose-dependent manner. Moreover, the wound healing assay showed that TQ treatment clearly inhibited the migration of 786-O-SI3 cells as compared to the control. Overall, the study indicated the ability of TQ to inhibit the cell migration and invasion of 786-O-SI3. In addition to that, TQ significantly inhibited the protein expression and gene transcription of MMP-2 and u-PA, which are the main reasons behind the highly invasive character of 786-O-SI3 cells. This MMP-2 downregulation was linked to the suppression of phosphoinositide 3-kinases (PI3K)/Akt and the Src/Paxillin signaling cascade. TQ also attenuated the cell adhesion to type I and type IV collagen. In vivo investigation of TQ in mice resulted in a significantly reduced number of 786-O-SI3 cells transferred to the lung. TQ administration attenuated the colonies of 786-O-SI3 cells in lung tissues and decreased the lung weights of xenografted mice, overall confirming an inhibitory effect on lung metastasis in vivo [36]. Very similar results were found in the same context of RCC but on two other cell lines (786-O and ACHN). TQ was shown to effectively inhibit the metastatic capacity of RCC cells in vitro, which was also verified in a xenograft model. TQ suppressed migration, invasion, and epithelial–mesenchymal transition in both cell lines and was shown to induce autophagy via the AMPK/mTOR signaling pathway. Interestingly, the observed inhibition of migration and invasion was autophagy-dependent, where the inhibition of autophagy attenuated TQ-induced anti-migratory effects [37].

These anti-metastatic properties are particularly relevant for aggressive cancers with poor prognosis, such as triple-negative breast cancer and renal cell carcinoma, where metastatic burden is the main cause of mortality. TQ’s inhibition of EMT, MMPs, and integrin-mediated adhesion further highlights its value as a metastasis-targeted agent.

### 2.4. Oxidative Stress and Inflammation Modulation

In the article by Shanmugam et al. mentioned before, the observed suppression of CXCR4 in MDA-MB-231 by TQ was thought to be transcriptionally regulated, as treatment also downregulated the nuclear factor kappa-light-chain-enhancer of activated B cells (NF-κB), possibly resulting in the suppression of NF-κB binding to the CXCR4 promoter. Downregulation of CXCR4 was further correlated with the inhibition of CXCL12-mediated migration and invasion of MDA-MB-231 cells [35]. Another study on breast cancer MDA-MB-468 and T47D cell lines revealed the ability of TQ to induce reactive oxygen species (ROS), leading to DNA damage, cell-cycle arrest, and apoptosis. The study showed that cancer cells with elevated antioxidant defenses, such as glutathione (GSH) and NQO1, were more resistant to TQ-induced oxidative stress. Notably, depletion of GSH sensitized the cells to TQ, enhancing its cytotoxic effects [38]. Furthermore, El-Najjar et al. showed that TQ treatment resulted in the generation of reactive oxygen species (ROS) and reduced the proliferation of cancer cells in a panel of human colon cancer cells (Caco-2, HCT-116, LoVo, DLD-1, and HT-29). Additionally, TQ induced apoptosis through the phosphorylation of JNK and ERK, and the activation of the MAPK pathway. Along with these effects, TQ did not exhibit cytotoxicity to normal human intestinal FHs74Int cells [39]. Similarly, TQ treatment led to a dose-dependent downregulation of NF-κB signaling in hepatocellular carcinoma HepG2 and Huh-7 cell lines. TQ was able to inhibit the growth of these cell lines through the generation of ROS, heme oxygenase-1 (HO-1), and NAD(P)H quinone dehydrogenase 1 (NQO1), as well as the inactivation of Bcl-2, IL-8, and their receptors [27]. This growth-impeding effect of TQ through ROS generation was also observed in C4-2B and PC-3 prostate cancer cell lines, where consequently, JNK was activated, leading to an increased modulation of GADD45α (DNA damage-inducible gene) and AIF (apoptosis-inducing factor-1), reduced regulation of Bcl-2-associated proteins, and, overall, prostate cancer cell death [21]. Interestingly, TQ appears to exploit redox imbalance as a vulnerability in cancer cells, triggering oxidative stress-induced apoptosis while sparing normal cells with intact antioxidant responses. These findings suggest that TQ’s modulation of oxidative stress pathways plays a crucial role in its anticancer activity.

### 2.5. Autophagy Modulation

Autophagy is considered a double-edged sword in cancer biology. While basal autophagy functions as a fundamental homeostatic mechanism involved in intracellular recycling and metabolic regulation, it is also activated in response to various stressors. Under such conditions, autophagy facilitates the clearance of damaged proteins and organelles, thereby promoting cellular stress tolerance, mitigating damage, and preserving cell viability. Although autophagy is recognized as a tumor-suppressive process in the early stages of oncogenesis, it can paradoxically support tumor cell survival under hostile microenvironmental conditions, such as hypoxia and nutrient deprivation [40]. TQ modulates autophagy in a context-dependent manner by acting on canonical nutrient- and stress-sensing pathways. In several carcinoma models, including renal cell carcinoma 786-O, ACHN, and SASVO3 head-and-neck cells, TQ activates AMPK, leading to downstream mTOR inhibition and increased LC3-II/Beclin-1 expression, consistent with autophagy induction, that culminates in autophagic cell death [37,41]. In colon carcinoma (LoVo) TQ activates JNK and p38 MAPK, causing caspase-independent autophagic cell death [42]. Conversely, in some glioblastoma models (U87MG, Gli36ΔEGFR), TQ inhibits autophagy (reduced Beclin-1 and ATG7 expression) and promotes lysosomal membrane permeabilization with cathepsin-B release, thereby sensitizing cells to temozolomide and favoring non-autophagic death pathways [43]. These dual effects indicate TQ’s action is determined by tumor lineage and metabolic state: activation of AMPK/mTOR or MAPK-driven autophagy tends to mediate cytotoxic autophagy in susceptible tumors, whereas autophagy inhibition and lysosomal destabilization can be leveraged where autophagy is a survival mechanism. Recognizing which pathway predominates in a given tumor will be essential for rational combination strategies with autophagy modulators.

### 2.6. Integrative Mechanistic Overview of Thymoquinone

TQ engages multiple stress-response pathways that converge on tumor cell death and therapy sensitization. A central theme is redox priming: by elevating ROS, TQ suppresses NF-κB signaling and lowers pro-metastatic CXCR4, thereby limiting migration and invasion in triple-negative breast cancer models [35].

This redox stress also intersects with ER stress and activation of UPR. In bladder cancer cells (T24, 253J), TQ increases the ER-stress markers GRP78 and CHOP, induces cytochrome-c release, and activates caspase-3, indicating ER-stress-mediated mitochondrial apoptosis [44]. In colon cancer models, TQ similarly links ROS generation to JNK/ERK activation and apoptosis while sparing normal intestinal epithelial cells [39]. Collectively, these findings support a model in which ROS functions upstream, contributing both to NF-κB suppression and to ER-stress-driven apoptotic signaling in responsive tumor types.

UPR signaling is also linked to autophagy regulation and cell-cycle control. In renal cell carcinoma, TQ activates AMPK, suppresses mTOR, and increases LC3 and Beclin-1 expression, leading to autophagy, which suppresses cell migration and invasion; importantly, inhibition of autophagy reverses these anti-metastatic effects [37]. In parallel, TQ can induce phase-specific cell-cycle arrest that aligns with apoptotic responses, including the G2/M arrest observed during radiosensitization in colorectal cancer models [45].

Hypoxia signaling serves as another key point of pathway integration. Under hypoxic conditions, TQ reduces HIF-1α protein levels and transcriptional activity by promoting its ubiquitin-proteasome-mediated degradation, disrupting hypoxia adaptation in renal cancer cells [46]. Likewise, in pancreatic cancer cells (PANC-1, AsPC-1, BxPC-3), TQ downregulates HIF-1α via PI3K/AKT/mTOR inhibition and enhanced ubiquitination. Because ROS, ER stress, AMPK–mTOR signaling, and HIF-1α pathways are interconnected, these findings support a model in which TQ disrupts hypoxia-driven survival programs while promoting apoptosis or cytotoxic autophagy, depending on tumor context. This integrated mechanism also aligns with TQ’s reported combination effects. In pancreatic cancer, TQ enhances gemcitabine activity by suppressing Notch1 and PI3K/AKT/mTOR signaling and increasing apoptosis, including in orthotopic models [47].

Finally, when autophagy functions as a survival mechanism, TQ can shift cells toward alternative death pathways. In glioblastoma models, TQ disrupts lysosomal membrane integrity and triggers cathepsin-B release, blocking autophagic flux and promoting non-autophagic cell death. This illustrates TQ’s context-dependent ability to redirect cell-death programs [43].

## 3. Thymoquinone and Endoplasmic Reticulum (ER) Stress in Cancer

### 3.1. The Role of UPR in Cancer Cells

One of the major hallmarks of cancer is impaired endoplasmic reticulum (ER) stress response, driven by factors in the tumor microenvironment (TME) such as hypoxia, ROS, and metabolic imbalances, which trigger ER stress and activate the unfolded protein response (UPR). To adapt to ER stress, cancer cells use prolonged activation of the UPR. This promotes tumor growth, immunological evasion, and treatment resistance. Although the three main UPR branches IRE1α, PERK, and ATF6 are essential for preserving cellular homeostasis in stressful situations, cancer cells commonly use them as a means of survival. Pharmacological inhibition of IRE1α has been demonstrated to lower tumor burden and improve therapeutic outcomes, while aberrant IRE1α signaling promotes tumor growth [3]. Similarly, the UPR chaperone GRP78 has been shown to be overexpressed in several cancers, where it was linked to resistance to chemotherapeutic drugs and radiotherapy [48]. GRP78 suppression increases drug sensitivity and causes cell death. Another important pathway is the ATF6 pathway which is also a reason behind poor prognosis when overexpressed. Its upregulation allows cells to survive under stress conditions, thus leading to chemoresistance. Moreover, functional studies show that ATF6 suppresses apoptosis while promoting cell motility and proliferation [49]. PERK signaling, although beneficial during the early stress response, also promotes immune evasion and cancer cell survival. The high expression of PERK was associated with a poor prognosis in kidney renal papillary cell carcinoma, lower grade glioma, breast invasive carcinoma, and thyroid carcinoma. Blocking this pathway was found to be beneficial in several models, including melanoma, where its blockage results in paraptosis and immunogenic cell death [50,51]. Persistent ER stress affects the tumor microenvironment by promoting immune evasion features through pathways such as XBP1-regulated lipid metabolism and hypoxia-induced stemness. Taken together, these findings underscore the dual role of UPR signaling in tumor biology and highlight its potential as a therapeutic target in cancer treatment. Additionally, the interplay between UPR pathways and oncogenic signaling cascades such as PI3K/Akt, MAPK, and STAT3 is emerging as a critical area of investigation, revealing how ER stress contributes to cancer cell survival, metabolic adaptation, and immune evasion. Understanding these network interactions may guide the development of combination therapies that disrupt compensatory survival mechanisms.

### 3.2. Thymoquinone as an ER Stress Modulator

A study investigated the effects of TQ on human bladder cancer cell lines T24 and 253J, focusing on its role in inducing apoptosis through ER stress-dependent mitochondrial pathways. The study showed that TQ treatment led to significant ER stress, evidenced by the upregulation of ER stress markers such as GRP78 and CHOP. This stress triggered mitochondrial dysfunction, resulting in the release of cytochrome c and activation of caspase-3, culminating in apoptosis. These findings suggest that TQ induces cell death in bladder cancer cells by activating ER stress pathways that lead to mitochondrial-mediated apoptosis [44]. Another article by Aslan et al. showed that TQ significantly increased GRP78 mRNA and protein levels while decreasing cell viability, S1P, C1P, NF-κB1 mRNA, and NF-κB p65 protein levels in MCF-7 and HepG2 cancer cells compared to controls. Moreover, it caused a significant increase in N-SMase activity, cellular levels of C16-C24 CERs, and cleaved caspase-3 levels. Overall, the article showed that TQ induces ceramide accumulation and ER stress and suppresses S1P, C1P, and NF-κB-mediated cell survival [52]. These findings highlight TQ’s capacity to disrupt cellular homeostasis via ceramide-mediated ER stress, suggesting a potential therapeutic window where TQ selectively induces lethal stress in malignant cells. However, the mechanistic link between TQ-induced ceramide accumulation and upstream UPR activation remains to be fully delineated.

Other than these studies, and to the best of our knowledge, there are no other articles discussing the role of TQ as an ER stress modulator in the context of cancer. However, there are some articles discussing its effects on ER stress in normal cells. For instance, in an in vitro excitotoxicity model using hippocampal neuronal cells, TQ demonstrated significant neuroprotective effects by modulating ER stress responses. Specifically, TQ reduced the expression of GRP78 and GRP94, and downregulated pro-apoptotic proteins such as BAX and cleaved PARP1, thereby attenuating ER stress-induced apoptosis [53]. Similarly, in a rat model of partial hepatic warm ischemia–reperfusion (I/R) injury, TQ was shown to alleviate ER stress and inhibit mitochondria-mediated apoptosis. TQ treatment significantly reduced the expression of ER stress markers such as GRP78 and CHOP, while also downregulating pro-apoptotic mitochondrial signals including cytochrome c and caspase-3 [54]. The contrasting roles of TQ in inducing ER stress in cancer cells versus alleviating ER stress in normal tissues underscore its context-dependent activity. This duality positions TQ as a unique candidate for selective cancer therapy with potentially reduced off-target toxicity. Future studies should aim to elucidate the molecular determinants that govern this selectivity.

### 3.3. Synergy with ER Stress-Inducing Agents

TQ was shown to overcome chemoresistance and potentiate the anticancer activity of bortezomib in a multiple myeloma xenograft mouse model. This synergistic effect was primarily mediated through the suppression of NF-κB signaling and its downstream anti-apoptotic and pro-survival gene products. The combination treatment significantly enhanced apoptosis and reduced tumor burden compared to monotherapy, suggesting that TQ may sensitize resistant cancer cells to proteasome inhibition via NF-κB pathway modulation [55].

Currently, there is no direct evidence in the scientific literature demonstrating that TQ synergizes with classical ER stress inducers such as tunicamycin or thapsigargin in cancer models. However, given TQ’s ability to induce ER stress and apoptosis in cancer cells, it is plausible that combining TQ with such ER stress inducers could result in synergistic anticancer effects. Thus, further research is needed to explore this potential combination therapy. Incorporating TQ into combination regimens with known ER stress inducers or immune checkpoint inhibitors may enhance therapeutic efficacy, particularly in tumors with a high UPR dependency. Investigating TQ’s impact on immunogenic cell death markers, such as calreticulin exposure and ATP release, may also provide insight into its potential to enhance anti-tumor immunity. Further studies are warranted to elucidate the dose-dependent effects of TQ on ER stress activation in relation to its cytotoxic outcomes. Clarifying this relationship will help distinguish whether ER stress serves primarily as an adaptive or pro-apoptotic signal at varying concentrations, thereby optimizing therapeutic applications.

## 4. Thymoquinone and Cancer Stem Cells (CSCs)

CSCs are well known for their resistance to conventional anticancer therapies and their critical role in tumor relapse. This resistance arises from several intrinsic features, including high proliferative capacity, self-renewal ability, angiogenic potential, and immune evasion. CSCs are typically identified by surface markers such as CD44, CD24, CD133, EpCAM, and LGR5, which vary across cancer types [56,57]. These cells exist within a tumor-promoting microenvironment enriched with cytokines like HGF, TNF-α, and IL-6, which support the dedifferentiation, invasion, and maintenance of stemness [58]. Resistance in CSCs is further driven by the activation of DNA damage response pathways, upregulation of xenobiotic-metabolizing enzymes, anti-apoptotic proteins, and drug-efflux transporters [59]. These defense mechanisms not only allow CSCs to survive initial therapy but also contribute to minimal residual disease and eventual tumor recurrence, underscoring the urgent need for agents that can effectively eliminate CSCs or impair their maintenance. In addition, dysregulated signaling pathways such as Wnt/β-catenin and PI3K/Akt promote survival and therapy resistance [60].

Emerging evidence shows that TQ can counteract these mechanisms. Ndreshkjana et al. (2019) [61] demonstrated that TQ, especially when combined with 5-fluorouracil, effectively targeted colorectal CSCs by suppressing the Wnt/β-catenin and PI3K/Akt pathways. This combination significantly reduced CD133^+^ cell populations and impaired spheroid formation, indicating a loss of stemness and tumorigenicity. These results highlight TQ’s dual role as both a chemosensitizer and a direct inhibitor of CSC-driven resistance [61]. This dual action is particularly relevant in refractory tumors where CSC populations drive heterogeneity and therapeutic escape. TQ’s capacity to disrupt both stemness signaling and survival pathways positions it as a compelling candidate for combination regimens.

Supporting this, Bashmail et al. (2018) [62] found that TQ enhanced the cytotoxicity of gemcitabine in breast cancer cells by inducing apoptosis and autophagy. Notably, it reduced the CD44^+^/CD24^−^ CSC population, suggesting improved therapeutic responsiveness [62].

Likewise, Ibiyeye and Zuki (2020) showed that co-delivery of doxorubicin and TQ via CaCO_3_ nanoparticles derived from cockle shells effectively downregulated CD44^+^/CD24^−^ markers, suppressed ALDH activity, and reduced CSC self-renewal and invasiveness [63]. The nanoparticle-based delivery system also improved TQ’s bioavailability and tumor targeting, illustrating how advanced drug delivery platforms can enhance the pharmacological efficacy of phytochemicals like TQ in targeting CSCs.

Jehan et al. (2020) [64] further demonstrated that TQ selectively targets hepatocellular carcinoma cells with minimal cytotoxicity to normal liver cells. When used with cisplatin or doxorubicin, it induced a strong pro-apoptotic effect, particularly in p53-deficient models, highlighting its value as a selective and potent adjuvant therapy [64]. This selectivity is critical in developing less-toxic regimens, especially for patients with p53-mutant or p53-null tumors, which are notoriously resistant to traditional therapies.

**Figure 3:**
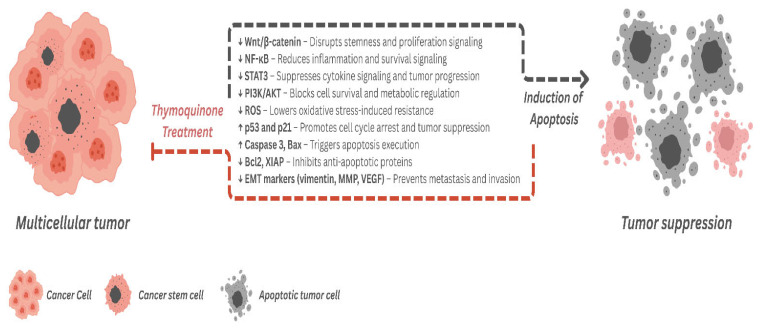
Mechanistic overview of thymoquinone’s action on cancer stem cells and tumor suppression. Thymoquinone exerts multi-targeted anticancer effects by modulating key signaling pathways involved in cancer stem cell (CSC) maintenance and tumor progression. TQ downregulates (↓) pathways that support stemness (Wnt/β-catenin), inflammation (NF-κB, STAT3), cell survival (PI3K/AKT), and metastasis (EMT markers such as vimentin, MMPs, and VEGF), while also reducing oxidative stress (ROS). Concurrently, it upregulates (↑) tumor suppressor proteins (p53, p21) and pro-apoptotic factors (Bax, Caspase-3) and inhibits anti-apoptotic regulators (Bcl-2, XIAP), thereby promoting apoptosis and tumor regression.

Overall, Figure 3 summarizes the mechanistic actions of TQ against CSCs, highlighting its modulation of critical signaling pathways, suppression of stemness-related markers, and induction of apoptotic cell death.

Although these findings are promising, further in vivo validation and mechanistic studies are required to determine the context-dependent effects of TQ on CSC plasticity, intratumoral heterogeneity, and resistance phenotypes. Clinical translation will also depend on optimizing delivery methods, bioavailability, and safety profiling in combinatorial settings.

## 5. Thymoquinone’s Effect on the Tumor Microenvironment

The tumor microenvironment (TME) plays a pivotal role in shaping cancer progression, metastasis, and therapeutic response. Beyond malignant cells, the TME comprises a dynamic network of stromal cells, immune cells, extracellular matrix components, and signaling molecules that support tumor growth and drug resistance. Thymoquinone (TQ), through its multi-targeted actions, has demonstrated the ability to disrupt various components of the TME, offering potential therapeutic benefit.

### 5.1. Why the TME Matters in Cancer Progression

#### Cancer-Associated Fibroblasts (CAFs), Macrophages (TAMs), and Immune Evasion

The TME is a multitude of proliferating non-neoplastic cells, including fibroblasts, macrophages, immune cells, and endothelial cells, that originate from the healthy neighboring tissues or are recruited from the circulation, and these ultimately contribute to carcinogenesis. The major stromal components in solid tumors are cancer-associated fibroblasts (CAFs) and tumor-associated macrophages (TAMs), both of which exert pro-tumorigenic functions. CAF and TAM are thought to modulate disease progression, therapy resistance, and clinical outcomes and may function in synergy, but the exact mechanism of their interaction remains poorly understood. CAF are the predominant cell type in the tumor stroma, and they play a role in cancer evolution and progression by contributing to the proliferative, pro-inflammatory, immunosuppressive, angiogenic, pro-invasive, and pro-metastatic TME [65]. Moreover, CAF drive the epithelial–mesenchymal transition (EMT), allowing cancer cells to gain motility and disseminate by reducing their polarity and adhesion. Similarly, TAMs produce growth factors and immunosuppressive cytokines that enhance tumor cells motility, intravasation, and invasion, and stimulate angiogenesis while preventing attacks by T cells and NK cells. Thereby the amount of infiltrating TAMs has a clinical correlation with tumor progression and reduced survival in cancer patients [66]. Targeting these stromal components has emerged as a promising strategy to overcome therapy resistance and reshape the immune landscape within tumors. Overall, the TME is being increasingly investigated in cancer research due to its role in driving evasion from immune surveillance and promoting rapid tumor growth and progression [14].

### 5.2. How Thymoquinone Modulates the TME

#### 5.2.1. Reprogramming TAMs from M2 (Pro-Tumor) to M1 (Anti-Tumor) Phenotype

TQ has been shown to inhibit angiogenesis by affecting two distinct cellular components of the TME. This immunomodulatory effect contributes to restoring anti-tumor immunity and reducing chronic inflammation in the tumor milieu. TQ downregulates the expression of VEGF and other angiogenic growth factors in cancer cells and inhibits the activation of signaling pathways downstream of VEGFR in tumor-associated endothelial cells [67]. The anti-angiogenic effect of TQ was also confirmed in vivo in a zebrafish angiogenesis model, where it suppressed intersegmental vessel formation in a dose-dependent manner and notably suppressed VEGF-A mRNA expression [68]. Moreover, TQ has profound anti-inflammatory activities, especially in macrophages, by inducing the suppression of inducible nitric oxide synthase (iNOS) upon oral administration in murine models that underwent inflammatory stimulation [69]. Similarly, in a ID8-NGL mouse model of ovarian cancer, TQ was reported to suppress NF-κB activity, decrease the population of M2 macrophages, and decrease soluble VEGF, reducing tumor cell proliferation and inducing apoptosis [70]. Furthermore, in triple-negative breast cancer (TNBC), TQ suppressed cell proliferation, migration, invasion, and metastasis by downregulating tumor-associated macrophage (TAM)-associated chemokine receptors [35]. Collectively, this indicates that TQ’s anticancer properties may be related to regulating the expression of TAM-associated proteins, highlighting its potential in both preventing and treating cancer.

#### 5.2.2. Suppression of CAFs and Reduction in Stromal Stiffness

CAFs, also referred to as myofibroblasts, are activated fibroblasts typically found in close proximity to cancer cells. They contribute to desmoplasia, a fibrotic response that increases tumor stiffness, impairs drug delivery, and fosters immune evasion. They can promote cancer progression and angiogenesis, reduce the dormancy of cancer cells, and hasten tumor growth. CAFs can secrete enzymes like lysyl oxidases and hydroxylases to catalyze the crosslinking of collagen to elastin and extracellular matrix molecules, attract vascular endothelial cells and monocytes from the bone marrow to enhance angiogenesis, and indirectly regulate vascularization and blood flow in tumors [14]. Recent studies show that TQ can suppress CAF activation and reduce the biochemical and mechanical properties of the fibrotic stroma. According to studies, TQ can suppress platelet-derived growth factor-BB (PDGF-BB), which lowers vascular smooth muscle cell migration and proliferation [71]. Additionally, TQ dose-dependently suppresses a number of growth factors in breast cancer cell lines in Bagg Albino (Balb/C) mice, including VEGF and EGF, which are the primary sources of CAF [71]. Further in vitro studies on MDA-MB-231 cancer stem cells confirmed TQ’s inhibitory effects on EGF and VEGF and showed its ability to inhibit Wnt3a and phosphatidylinositol-3 kinase (PI3K). This suggests that TQ’s cytoskeletal interference may directly impair the contractile and motile behavior of CAFs. In human astrocytoma cells and in Jurkat cells (T lymphoblastic leukemia cells) treated with TQ, degradation of α/β tubulin was noted, indicating a suppressive effect on CAFs, which rely heavily on cytoskeletal dynamics and microtubules made of α/β-tubulin for their motility, contractility, and ECM remodeling activities. These effects were also accompanied by an upregulation of tumor suppressor p73 and apoptosis [72].

#### 5.2.3. Downregulation of Hypoxia-Inducible Factors (HIF-1α)

TQ interferes with this hypoxia-driven oncogenic signaling cascade, thereby limiting tumor adaptation and aggressiveness. Hypoxia-inducible factors are the primary regulators of oxygen homeostasis, responsible for aligning oxygen supply with cellular demand throughout the human body. Cancer cells manipulate this homeostatic system to facilitate cancer progression. HIFs activate the transcription of thousands of genes that mediate angiogenesis, cancer stem cell specification, cell motility, EMT, extracellular matrix remodeling, glucose and lipid metabolism, immune evasion, invasion, and metastasis. This is clinically relevant as well where many advanced human cancers contain regions of intratumoral hypoxia where the median pO_2_ in cancers of the breast, cervix, and head/neck is 10 mmHg (~1.4% O_2_). This intratumoral hypoxia induces HIF-1α and HIF-2α protein expression, and an upregulation in the expression of one or both of these proteins is linked with increased patient mortality in a wide range of solid cancers and leukemias, as detected by immunohistochemical analysis of the diagnostic tumor biopsies [73]. Similar effects were observed upon TQ treatment in renal cancer cells, where TQ reduced HIF-1α protein levels, and this reduction effect was mediated via the ubiquitination-proteasome-dependent pathway. Furthermore, TQ inhibited HIF-1α transcriptional activities as it suppressed downstream genes of HIF-1α. TQ also resulted in anaerobic metabolic disturbance by altering glucose, lactate, and ATP levels, and induced apoptosis in hypoxic cancer cells [46]. These findings suggest that TQ could be particularly beneficial in targeting hypoxic niches often resistant to standard therapies.

Collectively, these findings highlight TQ’s potential in disrupting the tumor-supportive stroma, modulating hypoxic signaling, and enhancing therapeutic response. While promising, further research should focus on elucidating its impact on immune checkpoint regulation, stromal-immune crosstalk, and potential combinations with immune checkpoint inhibitors. Biomarkers such as tumor-infiltrating lymphocytes (TILs), microsatellite instability (MSI), tumor mutational burden (TMB), and angiogenic signatures should be investigated to predict and monitor TQ-mediated response.

## 6. Thymoquinone and Epigenetic Reprogramming in Cancer

In recent decades, growing evidence has highlighted the critical role of epigenetic modifications in cancer progression. Epigenetic alterations involve changes in gene expression without modifying the underlying DNA sequence [74]. The primary mechanisms involved include DNA methylation [75], histone modifications [76], and regulation by non-coding RNAs such as microRNAs, all of which modulate chromatin structure and gene accessibility. Disruption in these regulatory mechanisms can silence tumor suppressor genes or activate oncogenes, thus contributing significantly to tumor initiation, progression, and metastasis. Notably, unlike genetic mutations, epigenetic changes are potentially reversible, highlighting them as attractive therapeutic targets in cancer treatment [77].

### 6.1. Thymoquinone and Histone Modification

TQ exhibits promising potential in modulating histone acetylation and deacetylation processes in cancer cells. Primarily, TQ acts as an inhibitor of histone deacetylases (HDACs), enzymes frequently overexpressed in cancers such as MCF-7 breast cancer cells. HDACs play a key role in chromatin remodeling and are often upregulated in cancer, contributing to silencing of tumor suppressor genes. HDAC inhibition by TQ leads to increased histone acetylation, resulting in the reactivation of key tumor suppressor genes, including p21 and Maspin. This reactivation promotes apoptosis by elevating pro-apoptotic genes such as Bax and suppressing anti-apoptotic genes like Bcl-2, ultimately leading to cell-cycle arrest at the G2/M phase [78,79]. Additionally, TQ impacts the NAD^+^-dependent HDAC, SIRT1, which typically inhibits p53-mediated apoptosis. TQ enhances SIRT1 activity via the AMPK pathway, thereby regulating oxidative stress, inflammation, and apoptosis. Collectively, these findings underscore TQ’s therapeutic potential through histone modification pathways [80,81].

### 6.2. Thymoquinone as a DNA Hypomethylating Agent

TQ also plays a significant role in modulating DNA methylation, mainly by targeting DNA methyltransferase 1 (DNMT1). A study by Pang et al. demonstrated that TQ binds to the catalytic domain of DNMT1, where it competes with the methyl donor S-adenosylmethionine (SAM), or its by-product SAH (S-adenosylhomocysteine), thereby inhibiting enzymatic activity in a dose-dependent manner, with an IC_50_ of approximately 30 nM. Furthermore, TQ disrupts the Sp1/NF-κB complex at the DNMT1 promoter, decreasing DNMT1 expression and triggering global DNA hypomethylation. Consequently, leukemia cells exhibited reduced colony formation and increased apoptosis via caspase activation. In vivo, treatment with TQ led to regression of leukemia, decreased spleen size, and reduced leukemic infiltration in organs such as the lungs and liver. These outcomes indicate TQ’s capability as a natural hypomethylating agent with promising therapeutic value in leukemia [82]. Additionally, TQ-induced demethylation has shown therapeutic promise against chemoresistance. For example, in doxorubicin-resistant breast cancer cells (MCF-7/DOX), aberrant DNA methylation patterns contribute to suppressed expression of tumor suppressors such as PTEN, BRCA1, and RB1. TQ administration reversed these epigenetic changes by restoring PTEN expression, leading to cell-cycle arrest, mitochondrial membrane disruption, and apoptosis mediated by caspase-dependent PARP cleavage. Thus, TQ emerges as a novel epigenetic modulator capable of exerting anticancer effects via DNA hypomethylation [83]. These findings suggest that TQ may be particularly effective in epigenetically driven malignancies, especially those resistant to conventional chemotherapeutics.

### 6.3. Regulation of Non-Coding RNAs by Thymoquinone

MicroRNAs (miRNAs) are short non-coding RNAs that regulate gene expression post-transcriptionally, often acting as tumor suppressors or oncogenes. TQ also modulates non-coding RNAs, particularly miRNAs, involved in cancer progression. A study conducted by Imani et al. demonstrated that TQ enhances the tumor-suppressive functions of miR-34a, a microRNA known to suppress critical EMT-associated transcription factors, including Twist1 and Zeb1. In triple-negative breast cancer (TNBC) cells, the combined delivery of TQ and miR-34a markedly reduced EMT signaling, emphasizing TQ’s potential to enhance miR-34a’s therapeutic efficacy in controlling cancer metastasis [84]. Similarly, Meral et al. found that TQ treatment downregulated the oncogenic miR-206b-3p, thereby reducing oxidative stress, limiting necrosis, and suppressing tumor growth in vivo [85].

## 7. Thymoquinone in Combination Therapy: Enhancing Chemotherapy and Overcoming Drug Resistance

### 7.1. Thymoquinone + Chemotherapy

Despite the available chemotherapeutic agents in current use, prognosis remain poor and mortality rates are high at advanced stages of several cancers. This is mainly due to the chemoresistance encountered by these drugs, their non-specific cytotoxicity which results in bone marrow suppression, and other organ toxicity and collateral effects [62]. Thus, novel combination or adjuvant therapies are urgently needed to overcome these issues. There is ample evidence from preclinical studies showing that TQ can potentiate chemotherapeutic agents and significantly impede cancer progression. Studies indicate that TQ enhances chemosensitivity across multiple drug classes via overlapping but distinct mechanisms. For nucleoside analogs (e.g., gemcitabine), TQ pretreatment commonly reduces survival signaling (Notch1, PI3K/Akt/mTOR), increases apoptotic signaling (Bax/caspases), and remodels hypoxic/collagenous stroma to improve drug penetration and efficacy [47,86,87]. With anthracyclines (doxorubicin), TQ often augments mitochondrial apoptosis and may reduce cardiotoxicity when delivered in nano formulations [88]. Taxanes (paclitaxel/docetaxel) combinations frequently diminish tumor-associated stem-cell populations (CD44^+^/CD24^−^), decreasing resistant fractions and improving tumor control [47,89]. Proteasome inhibitors (bortezomib) showed NF-κB abrogation with restored drug sensitivity in multiple myeloma xenografts [90]. Across models, the principal functional improvements include increased tumor cell apoptosis and reduced clonogenicity, depletion of cancer stem-like cells and sphere-forming ability, radio sensitization via enhanced DNA damage/G2–M arrest, and in some nano-delivery contexts, mitigation of chemotherapy toxicity.

A representative, condensed summary of key preclinical combination studies investigating TQ with standard chemotherapeutics is presented in Table 1; the comprehensive list including full experimental details, detailed mechanisms, and limitations is provided in Supplementary Table S1.

### 7.2. Thymoquinone + Radiation Therapy

A study by Velho-Pereira et al. [93] assessed the radio-sensitizing potential of TQ on human breast carcinoma cells (MCF-7 and T47D). The study found that TQ in combination with a single dose of ionizing radiation (2.5 Gy) was able to exert supra-additive cytotoxic effects on both cell lines as measured by cell proliferation and colony-formation assays. This TQ-mediated radio-sensitization is thought to be related to apoptosis and cell cycle modulation [93]. Another study assessed the combination of TQ with ionizing radiation in two-dimensional (2D) and three-dimensional (3D) culture models derived from HCT116 and HT29 CRC cells, and in patient-derived organoids (PDOs). TQ was found to sensitize CRC cells to IR and reduce cell viability and clonogenic survival without inducing toxicity in normal intestinal cells. These sensitizing effects were linked with G2/M arrest and DNA damage. Moreover, this combination was able to fully inhibit sphere formation and inhibit stemness and DNA repair. It also decreased organoid forming ability of PDOs [45]. Collectively, these findings underscore TQ’s potential as a radiosensitizer that selectively targets cancer cells without harming normal tissue.

### 7.3. Thymoquinone + Immunotherapy (PD-1/PD-L1 Blockade)

A clinical report was the first of its type to show the clinical efficacy of combined TQ (oral capsules) with dual immune checkpoint inhibitors (ICPIs) nivolumab plus ipilimumab, which act via CTLA-4/PD-1 blockade, in patients with metastatic extra-pulmonary neuroendocrine neoplasms (EP-NECs) who were refractory to first-line chemotherapy. The study demonstrated durable responses, with progression-free survival exceeding two years. Notably, none of the patients experienced significant toxicity, and all reported improvements in quality of life [94]. To the best of our knowledge, this is the only study that investigates the combination of TQ with immunotherapy.

Further clinical investigations are warranted to validate these findings and elucidate the molecular underpinnings of TQ’s immunomodulatory properties.

## 8. Challenges and Future Research Directions

While preclinical studies provide strong evidence of thymoquinone’s (TQ) anticancer activity, several factors hinder clinical translation. A key obstacle is poor bioavailability due to low aqueous solubility, rapid metabolism, and high systemic clearance [95]. Advanced delivery systems (nanoparticles, nanostructured lipid carriers, PEGylated, and liposomal formulations) are being developed to improve its stability, exposure, tumor targeting, and biodistribution; notably, TQ-loaded PLGA nanoparticles and lipid-based carriers have enhanced pharmacokinetic profiles and efficacy in preclinical models. A second limitation is the absence of large clinical trials evaluating safety, pharmacokinetics, and therapeutic benefit, either alone or in combination with standard therapies. Finally, TQ’s pleiotropic actions (apoptosis, autophagy, oxidative stress, inflammation, epigenetic regulation) are context-dependent, arguing for tumor-specific strategies that consider interindividual variability in metabolism and transport, as well as tumor signaling profiles. Integrating pharmacogenomics with biomarker-guided patient selection may enable a precision-medicine approach.

## 9. Clinical and Translational Potential

We recommend dose–exposure–response studies linking plasma and tumor levels to pharmacodynamic readouts (for example, p65 nuclear localization, CHOP, LC3-II, γH2AX, HIF-1α) using LC–MS/MS and population PK/PD modeling; head-to-head optimization of nano-delivery systems (liposomes, polymeric nanoparticles such as PLGA, micelles, SLN/NLC) against a target product profile prioritizing exposure, stability, release kinetics, tumor uptake, and safety; mechanism-guided combinations, such as gemcitabine in pancreatic models with active PI3K/AKT/mTOR and Notch signaling, bortezomib in NF-κB-dependent myeloma, and radiotherapy in colorectal models showing G2/M arrest, and early clinical studies (Phase 0/1) with intensive PK, mechanism-anchored biomarkers, food-effect and drug–drug interaction assessments, and exposure–response analyses to guide dosage, schedule, and patient selection.

## 10. Conclusions

Preclinical studies consistently show that thymoquinone induces apoptosis and modulates oxidative stress, including ROS-linked suppression of survival signaling such as NF-κB. These are the most robust and reproducible mechanisms across tumor models. A second tier of support includes ER stress and unfolded protein response (UPR)-associated apoptosis, phase-specific cell-cycle arrest, and hypoxia/HIF-1α attenuation, which are biologically coherent but less broadly profiled. Autophagy modulation is context-dependent, sometimes cytotoxic and sometimes pro-survival. Epigenetic effects (HDAC/DNMT, microRNAs) and cancer-stem-cell targeting are promising but remain exploratory, with limited in vivo replication. Together, these mechanisms help explain reported chemo- and radio-sensitizing effects.

Key next steps are standardized in vivo validation; pharmacokinetic, bioavailability, and formulation studies; clarification of when to inhibit or leverage autophagy; biomarker-guided combination strategies (for example, ROS or UPR activity, NF-κB status, hypoxia signatures); and early-phase clinical studies with mechanism-anchored endpoints.

## Figures and Tables

**Table 1:** Summary of key preclinical studies demonstrating Thymoquinone’s ability to potentiate chemotherapeutic agents across various cancer types. N/Y stands for No or Yes for in vivo investigation.

Study	Chemotherapy	Model (In Vitro/In Vivo)	Key Outcome	Primary Mechanism	In Vivo?
Bashmail et al., 2018 [62]	Gemcitabine	MCF-7, T47D	TQ potentiated gemcitabine cytotoxicity	↑apoptosis, ↑autophagy markers	N
Bashmail et al., 2020 [90]	Paclitaxel	MCF-7, T47D	Reduced PTX-resistant fraction; depleted CSCs	CSC marker downregulation (CD44^+^/CD24^–^)	N
Dirican et al., 2015 [88]	Docetaxel	DU-145	Synergistic cytotoxicity and apoptosis	PI3K–AKT pathway modulation	N
Effenberger-Neidnicht and Schobert, 2011 [87]	Doxorubicin	Multiple cell lines (+ MDR variants)	Enhanced growth inhibition in select lines	Mitochondrial apoptosis, ↑ROS, caspase activation	N
Mu G gang et al., 2016 [47]	Gemcitabine	PANC-1; orthotopic xenograft	Pretreatment with TQ enhanced GEM antitumor effect	Notch1 and PI3K/Akt/mTOR suppression	Y
El-Far AH et al., 2021 [91]	Doxorubicin	HCT116, MDA-MB-231; xenografts	Tumor growth inhibition + reduced DOX cardiotoxicity	↑Bax, ↓Bcl-2; improved PK via nanoformulation	Y
Siveen KS et al., 2014 [55]	Bortezomib	MM cell lines; xenograft	Overcame resistance; reduced tumor burden	NF-κB pathway abrogation	Y
Khalife R et al., 2014 [92]	Topotecan	U937 (AML)	Synergistic antiproliferative and proapoptotic effects	↑Bax/p53, caspase activation	N

↑ indicates an increase in the mentioned process, while ↓ indicates a decrease in the process or decreased expression.

## Data Availability

No new data were created or analyzed in this study. Data sharing is not applicable to this article.

## Supplementary Materials

The following supporting information can be downloaded at: https://www.mdpi.com/article/10.3390/ijms262211029/s1. References [96,97,98,99,100,101] are cited in the supplementary materials.

## Abbreviations

The following abbreviations are used in this manuscript:

Akt	Protein kinase B
ATP	Adenosine triphosphate
CSC	Cancer stem cell
CXCR4	C-X-C chemokine receptor type 4
DNMT1	DNA cytosine-5-methyltransferase 1
DOX	Doxorubicin
ECM	Extracellular matrix
EMT	Epithelial–mesenchymal transition
ER	Endoplasmic reticulum
ERK	Extracellular signal-regulated kinase
IC_50_	Half-maximal inhibitory concentration
LC3	Microtubule-associated protein 1A/1B-light chain 3
MAPK	Mitogen-activated protein kinase
NF-κB	Nuclear factor kappa-light-chain-enhancer of activated B cells
PCNA	Proliferating cell nuclear antigen
PD	Pharmacodynamics
PDAC	Pancreatic ductal adenocarcinoma
PDGF-BB	Platelet-derived growth factor-BB
PI3K	Phosphoinositide 3-kinase
RCC	Renal cell carcinoma
ROS	Reactive oxygen species
SAM	S-adenosylmethionine
TAM	Tumor-associated macrophage
