# Supplementary material for: Targeting Cancer Through Thymoquinone: From Molecular Mechanisms to Clinical Prospects
Source: Int J Mol Sci. 2025 Nov 14;26(22):11029. doi: 10.3390/ijms262211029 (PMC12652967; doi:10.3390/ijms262211029)
Supplement: Supplementary file 1 [file ijms-26-11029-s001.zip › ijms-3967536-supplementary.docx]

Table S1. Summary of key preclinical studies demonstrating Thymoquinone’s ability to potentiate chemotherapeutic agents across various cancer types.

| **Study** | **Chemotherapy** | **Model** | **Key findings** | **Mechanism** | **Limitation** |
| --- | --- | --- | --- | --- | --- |
| Thymoquinone synergizes gemcitabine anti-breast cancer activity via modulating its apoptotic and autophagic activities [1] | Gemcitabine (GCB) | MCF-7 and T47D | TQ improves the cytotoxicity of GCB in breast cancer cell lines | increasing the Pre-G cell population, apoptosis and autophagy markers | Lack of in vivo validation |
| Thymoquinone Enhances Paclitaxel Anti-Breast Cancer Activity via Inhibiting Tumor-Associated Stem Cells Despite Apparent Mathematical Antagonism [2] | Paclitaxel (PTX) | MCF-7 and T47D | TQ reduced PTX-resistant cell fractions and enhanced cell death, despite showing mathematical antagonism in combination index analyses. | TQ depleted tumor-associated stem cells (CD44⁺/CD24⁻), downregulated TWIST-1, upregulated SNAIL1/2, and induced apoptosis in T47D cells and autophagic cell death in MCF-7 cells | Lack of in vivo validation |
| Thymoquinone Augments Cyclophosphamide-Mediated Inhibition of Cell Proliferation in Breast Cancer Cells[3] | Cyclophosphamide (CPA) | MCF-7 and MDA-MB-231 | TQ enhanced the antiproliferative effect of CPA in a dose-dependent manner, significantly reducing cell viability compared to either agent alone. | The combination treatment induced greater apoptosis, decreased oxidative stress markers, and modulated antioxidant enzyme activity, suggesting a role for ROS generation in promoting cell death | Several molecular pathways are underinvestigated |
| Synergistic Role of Thymoquinone on Anticancer Activity of 5-Fluorouracil in Triple Negative Breast Cancer Cells [4] | 5-Fluorouracil (5-FU) | MDA-MB-231 and BT-549 | TQ significantly enhanced the cytotoxic and antiproliferative effects of 5-FU | The combination induced apoptosis via caspase-3 activation, ROS generation, mitochondrial membrane potential loss, and suppression of anti-apoptotic Bcl-2 protein, pointing to a mitochondrial-dependent apoptotic pathway. | Lack of in vivo validation |
| Targeted Apoptotic Effects of Thymoquinone and Tamoxifen on XIAP Mediated Akt Regulation in Breast Cancer[5] | Tamoxifen | MCF-7 and T47D | The combination of TQ and tamoxifen resulted in a synergistic reduction in cell viability, enhanced apoptosis, and greater suppression of survival signaling compared to either agent alone | The combination downregulated XIAP (X-linked inhibitor of apoptosis protein), leading to decreased Akt phosphorylation and induction of mitochondrial apoptosis through cytochrome c release and caspase activation. | Lack of in vivo validation |
| The combination of thymoquinone and paclitaxel shows anti-tumor activity through the interplay with apoptosis network in triple-negative breast cancer [6] | Paclitaxel | MDA-MB-231, MDA-MB-468 | TQ enhanced the anti-tumor efficacy of paclitaxel by increasing apoptosis and reducing tumor volume more effectively than either agent alone. | The combination modulated apoptosis by upregulating pro-apoptotic markers (Bax, cleaved caspase-3) and downregulating anti-apoptotic proteins (Bcl-2, survivin), thus promoting mitochondrial-mediated apoptosis. | Lack of in vivo validation |
| Thymoquinone Could Increase The Efficacy of Tamoxifen Induced Apoptosis in Human Breast Cancer Cells: An In Vitro Study.[7] | Tamoxifen | MCF-7,MDA-MB-231 | TQ enhanced the apoptotic effect of tamoxifen in both cell lines, with increased cell death observed compared to tamoxifen alone | Combination treatment increased apoptotic cell population (TUNEL and AO/EB staining), and reduced viability (MTT assay). | Lacks mechanistic exploration at molecular signaling level |
| Combinatorial effects of thymoquinone on the anti-cancer activity of doxorubicin[8] | Doxorubicin | HL-60 (leukemia), 518A2 (melanoma), HT-29 (colon), KB-V1 (cervix), MCF-7 (breast carcinoma), and their multidrug-resistant variants, and non-malignant human fibroblasts (HF) | TQ enhanced the anti-cancer efficacy of doxorubicin in a cell line-specific manner. Notably, the combination significantly increased growth inhibition in HL-60 and multidrug-resistant MCF-7/TOPO cells compared to doxorubicin alone. | The combination therapy induced apoptosis through multiple pathways: increased DNA fragmentation, elevated activities of caspases-3, -8, and -9, disruption of mitochondrial membrane potential, and an increased Bax/Bcl-2 mRNA expression ratio. Additionally, there was a higher generation of reactive oxygen species (ROS) in HL-60 cells with the combination treatment | An overall comprehensive study but lacks in vivo validation |
| Thymoquinone Pretreatment Overcomes the Insensitivity and Potentiates the Antitumor Effect of Gemcitabine Through Abrogation of Notch1, PI3K/Akt/mTOR Regulated Signaling Pathways in Pancreatic Cancer [9] | Gemcitabine | PANC-1, in vivo: orthotopic xenograft | pretreatment significantly enhanced gemcitabine-induced apoptosis and tumor growth inhibition in both in vitro and in vivo models, and led to synergistic increase in cancer cell apoptosis and reduction in tumor volume compared to either agent alone | enhanced antitumor effect was associated with suppression of Notch1 signaling and its intracellular domain (NICD), upregulation of PTEN expression, inactivation of the PI3K/Akt/mTOR/S6 signaling pathway, inhibition of NF-κB activation and its downstream anti-apoptotic proteins (Bcl-2, Bcl-xL, XIAP), and activation of pro-apoptotic molecules including Bax, caspase-3, caspase-9, and increased cytochrome c | Comprehensive study, optimal dosing regimen should be determined in clinical trials |
| Thymoquinone affects the gemcitabine sensitivity of pancreatic cancer by regulating collagen via hypoxia inducible factor-1α [10] | Gemcitabine (GEM) | PANC-1 cell line and corresponding animal model | The combination of TQ and GEM was more effective than GEM alone in inhibiting migration and invasion and promoting apoptosis | Downregulation of hypoxia-inducible factor-1α (HIF-1α), decreased expression of collagen types I, III, and V (COL1A1, COL3A1, COL5A1), and inhibition of transforming growth factor-β1 (TGFβ1)/Smad signaling pathway | The study used a single pancreatic cancer cell line (PANC-1) and animal model but did not employ any other cell line for validating and generalizing the results |
| Thymoquinone and its Nanoformulation Attenuate Colorectal and Breast Cancers and Alleviate Doxorubicin-Induced Cardiotoxicity [11] | Doxorubicin (DOX) | HCT116, MDA-MB-231-Luc, in vivo model: xenograft tumor models in mice | TQ and its nanoformulation, both alone and in combination with DOX, significantly inhibited tumor growth in colorectal and breast cancer models. Additionally, the combination treatments markedly reduced DOX-induced cardiotoxicity in mice. | Upregulation of pro-apoptotic protein Bax in HCT116 cells, downregulation of anti-apoptotic protein Bcl-2 in MDA-MB-231-Luc cells, enhanced induction of apoptosis, and overall reduction in tumor size in xenograft models | Only one cell line per cancer type was employed for testing. |
| The effects of thymoquinone and Doxorubicin on leukemia and cardiomyocyte cell lines [12]. | Doxorubicin (DOX) | Human leukemia cell line (RAW) and cardiomyocyte cell line | The combination of TQ and DOX led to enhanced antiproliferative effects in leukemia cells and reduced cytotoxic effects in cardiomyocyte cells suggesting that TQ may potentiate the anticancer activity of DOX while mitigating its cardiotoxic side effects | RAW cell apoptosis increased in combination therapy with TQ. Combination treatment also demonstrated significant cardiac myocyte survival at concentrations when used alone were able to reduce the leukemia cells | Lacks in vivo validation |
| Thymoquinone upregulates miR‐125a‐5p, attenuates STAT3 activation, and potentiates doxorubicin antitumor activity in murine solid Ehrlich carcinoma [13]. | Doxorubicin | In vivo murine solid Ehrlich carcinoma model | TQ enhanced the antitumor efficacy of DOX in mice bearing solid Ehrlich tumors. The combination therapy resulted in greater tumor growth inhibition compared to either agent alone | Upregulation of tumor suppressor microRNA-125a-5p, suppression of STAT3 phosphorylation without affecting STAT5, downregulation of STAT3 downstream target proteins, and increased apoptotic activity via activation of caspase-3 and -9 | Further research is needed to confirm these findings in human clinical settings. |
| Thymoquinone Could Increase The Efficacy of Tamoxifen Induced Apoptosis in Human Breast Cancer Cells: An In Vitro Study [7]. | Tamoxifen (TAM) | MDA-MB-231, MCF7 | The combination of TQ and TAM significantly reduced cell viability and increased apoptosis in both cell lines compared to individual treatments. | The study demonstrated that the combined treatment led to increased apoptotic index as evidenced by TUNEL assay and AO/EB staining. Morphological changes characteristic of apoptosis were also observed. | Lacks in vivo validation |
| Thymoquinone overcomes chemoresistance and enhances the anticancer effects of bortezomib through abrogation of NF-κB regulated gene products in multiple myeloma xenograft mouse model [14]. | Bortezomib | Human multiple myeloma (MM) cell lines (including drug-resistant variants) and CD138⁺ cells from MM patients’ in vivo model: MM xenograft mouse model | TQ inhibited proliferation of MM cells, including those resistant to doxorubicin, melphalan, or bortezomib. TQ also enhanced the apoptotic effects of bortezomib in MM cell lines. In the xenograft mouse model, the combination of TQ and bortezomib significantly reduced tumor volume compared to either agent alone | TQ activated caspase-3, leading to PARP cleavage and apoptosis, abrogated NF-κB signaling by downregulating p65 and its downstream targets, including Bcl-2 and VEGF, and inhibited CXCL12-induced chemotaxis and invasion in MM cells. | clinical trials are necessary to confirm efficacy and safety of such combination |
| Novel combination of docetaxel and thymoquinone induces synergistic cytotoxicity and apoptosis in DU-145 human prostate cancer cells by modulating PI3K–AKT pathway [15]. | Docetaxel | Human prostate cancer cell line DU-145 | The combination resulted in significant synergistic cytotoxicity and apoptosis in DU-145 cells compared to either agent alone | The combination treatment modulated the PI3K/Akt signaling pathway, leading to enhanced apoptosis. | Lack of in vivo studies |

References

1. Bashmail HA, Alamoudi AA, Noorwali A, Hegazy GA, AJabnoor G, Choudhry H, et al. Thymoquinone synergizes gemcitabine anti-breast cancer activity via modulating its apoptotic and autophagic activities. Sci Rep. 2018 Aug 3;8(1):11674.

2. Bashmail HA, Alamoudi AA, Noorwali A, Hegazy GA, Ajabnoor GM, Al-Abd AM. Thymoquinone Enhances Paclitaxel Anti-Breast Cancer Activity via Inhibiting Tumor-Associated Stem Cells Despite Apparent Mathematical Antagonism. Molecules. 2020 Jan 20;25(2):426.

3. Khan A, Aldebasy YH, Alsuhaibani SA, Khan MA. Thymoquinone Augments Cyclophosphamide-Mediated Inhibition of Cell Proliferation in Breast Cancer Cells. Asian Pacific Journal of Cancer Prevention. 2019 Apr 1;20(4):1153–60.

4. Zheng M, Mei Z, Junaid Md, Tania M, Fu J, Chen HC, et al. Synergistic Role of Thymoquinone on Anticancer Activity of 5-Fluorouracil in Triple Negative Breast Cancer Cells. Anticancer Agents Med Chem. 2022 Apr;22(6):1111–8.

5. Rajput S, Kumar BNP, Sarkar S, Das S, Azab B, Santhekadur PK, et al. Targeted Apoptotic Effects of Thymoquinone and Tamoxifen on XIAP Mediated Akt Regulation in Breast Cancer. PLoS One. 2013 Apr 17;8(4):e61342.

6. Şakalar Ç, İzgi K, İskender B, Sezen S, Aksu H, Çakır M, et al. The combination of thymoquinone and paclitaxel shows anti-tumor activity through the interplay with apoptosis network in triple-negative breast cancer. Tumor Biology. 2016 Apr 26;37(4):4467–77.

7. Ganji-Harsini S, Khazaei M, Rashidi Z, Ghanbari A. Thymoquinone Could Increase The Efficacy of Tamoxifen Induced Apoptosis in Human Breast Cancer Cells: An In Vitro Study. Cell J. 2016;18(2):245–54.

8. Effenberger-Neidnicht K, Schobert R. Combinatorial effects of thymoquinone on the anti-cancer activity of doxorubicin. Cancer Chemother Pharmacol. 2011 Apr 26;67(4):867–74.

9. Mu G gang, Zhang L li, Li H yan, Liao Y, Yu H gang. Thymoquinone Pretreatment Overcomes the Insensitivity and Potentiates the Antitumor Effect of Gemcitabine Through Abrogation of Notch1, PI3K/Akt/mTOR Regulated Signaling Pathways in Pancreatic Cancer. Dig Dis Sci. 2015 Apr 26;60(4):1067–80.

10. Zhao Z, Liu L, Chen H, Li S, Guo Y, Hou X, et al. Thymoquinone affects the gemcitabine sensitivity of pancreatic cancer by regulating collagen via hypoxia inducible factor-1α. Front Pharmacol. 2023 May 31;14.

11. El-Far AH, Salaheldin TA, Godugu K, Darwish NH, Mousa SA. Thymoquinone and its Nanoformulation Attenuate Colorectal and Breast Cancers and Alleviate Doxorubicin-Induced Cardiotoxicity. Nanomedicine. 2021 Jul 16;16(17):1457–69.

12. Brown RK, Wilson G, Tucci MA, Benghuzzi HA. The effects of thymoquinone and Doxorubicin on leukemia and cardiomyocyte cell lines. Biomed Sci Instrum. 2014;50:391–6.

13. Atteia HH, Arafa MH, Mohammad NS, Amin DM, Sakr AT. Thymoquinone upregulates miR‐125a‐5p, attenuates STAT3 activation, and potentiates doxorubicin antitumor activity in murine solid Ehrlich carcinoma. J Biochem Mol Toxicol. 2021 Dec 4;35(12).

14. Siveen KS, Mustafa N, Li F, Kannaiyan R, Ahn KS, Kumar AP, et al. Thymoquinone overcomes chemoresistance and enhances the anticancer effects of bortezomib through abrogation of NF-κB regulated gene products in multiple myeloma xenograft mouse model. Oncotarget. 2014 Feb 15;5(3):634–48.

15. Dirican A, Atmaca H, Bozkurt E, Erten C, Karaca B, Uslu R. Novel combination of docetaxel and thymoquinone induces synergistic cytotoxicity and apoptosis in DU-145 human prostate cancer cells by modulating PI3K–AKT pathway. Clinical and Translational Oncology. 2015 Feb 25;17(2):145–51.
